# Avoiding Tissue Overlap in 2D Images: Single-Slice DBT Classification Using Convolutional Neural Networks

**DOI:** 10.3390/tomography9010032

**Published:** 2023-02-14

**Authors:** João Mendes, Nuno Matela, Nuno Garcia

**Affiliations:** 1 Faculdade de Ciências, Instituto de Biofísica e Engenharia Biomédica, Universidade de Lisboa, 1749-016 Lisboa, Portugal; 2 Faculdade de Ciências, LASIGE, Universidade de Lisboa, 1749-016 Lisboa, Portugal

**Keywords:** carcinoma of the breast, CNN, DBT, deep learning

## Abstract

Breast cancer was the most diagnosed cancer around the world in 2020. Screening programs, based on mammography, aim to achieve early diagnosis which is of extreme importance when it comes to cancer. There are several flaws associated with mammography, with one of the most important being tissue overlapping that can result in both lesion masking and fake-lesion appearance. To overcome this, digital breast tomosynthesis takes images (slices) at different angles that are later reconstructed into a 3D image. Having in mind that the slices are planar images where tissue overlapping does not occur, the goal of the work done here was to develop a deep learning model that could, based on the said slices, classify lesions as benign or malignant. The developed model was based on the work done by Muduli et. al, with a slight change in the fully connected layers and in the regularization done. In total, 77 DBT volumes—39 benign and 38 malignant—were available. From each volume, nine slices were taken, one where the lesion was most visible and four above/below. To increase the quantity and the variability of the data, common data augmentation techniques (rotation, translation, mirroring) were applied to the original images three times. Therefore, 2772 images were used for training. Data augmentation techniques were then applied two more times—one set used for validation and one set used for testing. Our model achieved, on the testing set, an accuracy of 93.2% while the values of sensitivity, specificity, precision, F1-score, and Cohen’s kappa were 92%, 94%, 94%, 94%, and 0.86, respectively. Given these results, the work done here suggests that the use of single-slice DBT can compare to state-of-the-art studies and gives a hint that with more data, better augmentation techniques and the use of transfer learning might overcome the use of mammograms in this type of studies.

## 1. Introduction

The prevalence of breast cancer (BC) has been growing for several years and, in 2020, it became the most commonly diagnosed type of cancer [1]. The nefarious effects of this disease also represent a significant weight in the cancer paradigm: one in six deaths by cancer in women is caused by BC [2].

The emergence of breast cancer is related to both genetic predisposition (BRCA1 and BRCA 2 gene mutations put women at higher risk [3]) and environmental risk factors. Age and increased breast density are two of the most studied factors that contribute to the risk of developing this disease [4]. Several studies related dense breast patterns with an increased risk of BC [5].

One of the best weapons to fight the potential effects of breast cancer is to have an early diagnosis, which is currently aimed through generalized screening programs. Most of the worldwide screening programs use mammography as the standard imaging technique; however, its benefits and harms have been a topic of discussion in the scientific community [6].

The probability, in Europe, of a woman of age 50–69 with a biennial screening having a false positive result is 20%. On the other hand, in the United States of America, 50% of women will suffer the consequences of a false positive. Nonetheless, getting a false negative is also a possible outcome of screening with mammography: 28–33% of the cancers detected in women that undergo screening are interval cancer [6].

Since mammography is a two-dimensional image of the breast—which is a 3D volume—there will be tissue overlapping [7]. This fact can, consequently, lead to both tumour masking or false lesions appearance, hence contributing to false positive/negative rates. One way to surpass the barriers imposed by overlapping tissue is to use digital breast tomosynthesis (DBT). The fact that the DBT slices are so thin is what guarantees that the problem with overlapping tissue does not occur in DBT in the same way that it appears in mammography. Despite the fact that a DBT exam consists of several slice acquisitions, the dose of radiation is similar to that used in common mammography routines [8].

Nonetheless, it is important to verify how DBT compares with full-field digital mammography (FFDMG) in several other parameters. The use of DBT alongside FFDMG compared to the use of FFDMG alone increases the cancer detection rate [9]. These results should be looked upon carefully; even though detection of more cancers, and earlier, might positively impact women’s lives (less aggressive treatment and better outcomes), it is important to assure that this increase in detection rate is not related to overdiagnosis [10]. The use of DBT+FFDMG also has positive impacts in recall rates, which results in a higher specificity compared to the use of FFDMG alone [10].

Therefore, given that DBT alone can overcome the problem of tissue overlapping and that its use in clinical practice helps to improve the detection rate while decreasing recall rates, this study focuses on the use of DBT.

Artificial intelligence (AI) has made its way into medical diagnosis and more specifically into the field of breast cancer imaging. A review of several applications of AI to breast imaging, done by our team, can be found elsewhere [11]. Given that, the classification of DBT images into healthy/diseased classes or into benign/malignant lesions can also be done through AI.

A group of researchers [12] aimed to classify DBT images and whole mammograms using convolutional neural networks (CNN). In order to do that, they used both well-established algorithms—AlexNet [13] and ResNet [14]—and self-developed models. Different variations of the established models were used depending on the type of classification being made: 3D models if they wanted to classify DBT, and 2D models if the goal was to classify mammograms. Moreover, the authors also aimed to compare model performance for the AlexNet and ResNet with and without transfer learning [15].

The idea of combining the information of 2D mammograms and 3D DBT was proposed by Liang et al. [16]. There, 2D models were used both for mammogram and DBT classification. In order to do that, the authors did not use the entire volume to make a classification but rather extracted a “fixed slice” from the volume, which aimed to represent the observed changes across the different slices. Their model could be divided into a “backbone” architecture—a fully convolutional network (AlexNet, ResNet, SqueezeNet, or DenseNet) that served as a feature extractor—and an ensemble of classifiers, each one composed of a convolutional layer succeeded by two fully connected layers. There were three classifiers: one that classified the features extracted from mammography images, another that classified the features driven from DBT, and a third classifier that was focused on classifying the concatenation of the features extracted from each imaging modality. To decide the final output, a majority voting algorithm was applied.

The concept of retrieved fixed slices or dynamic images from a DBT volume has been used in other studies with the rationale of diminishing the computational burden that is associated with big 3D volumes. Zhang et al. [17] aimed to implement that and compare it with an alternative methodology where features were extracted from each slice and a final feature map was obtained by a pooling procedure across the different feature maps. Besides comparing these methods between themselves, the authors also compared both their methodologies with classic AlexNet architectures for 3D volumes. The authors found that any of the variations of their proposed methodology outperformed the classical architectures, which achieved 0.63 as the maximum AUC value. With their approach, they found out that the extraction of features from each slice and further pooling them into a final feature map produced better results than using the dynamic image; the maximum AUC values for each approach were 0.854 and 0.792, respectively, both using AlexNet architectures.

Furthermore, there are several different researches that aim to differentiate benign and malignant lesions. The work by Muduli et al. [18] that served as motivation for this research, aimed to classify lesions present in mammograms and ultrasound. To do that, the authors applied classic data augmentation techniques (translation, rotation, etc.) and used a CNN model with five learnable layers. Another group of researchers [19], to avoid the inflexible receptive field of 3D convolutions for DBT classification, developed a hybrid model that was able to extract hierarchical feature representations using 2D convolution in a slice-by-slice approach. Moreover, a self-attention module was used to learn structural representations of benign and malignant lesions. The idea of combining 2D and 3D convolution was also pursued by Xiao et al. [20] for slice feature extraction and lesion structural information, respectively. While many works define a region of interest to analyse the lesions, some authors [21] aim to use entire DBT slices for identifying the presence of lesions through the use of self-built CNN.

As it can be seen, there are several studies that aim to use the advantages given by DBT in relation to mammography, while maintaining the 2D convolution approach. These approaches allow the authors to overcome the problem of overlapping tissue, leading to lesion masking or fake lesion creation, while maintaining the low computational burden that is associated with 2D CNN architectures.

Given that, the aim of this work was to develop a CNN model to differentiate malignant lesions from benign lesions, using single DBT slices.

## 2. Materials and Methods

### 2.1. Dataset

The data used in this study came from the Breast Cancer Screening-Digital Breast Tomosynthesis (BCS-DBT) dataset, publicly available in the Cancer Imaging Archive [22]. This dataset contains DBT images from healthy, actionable, benign, and malignant cases. The cases were collected at Duke Health [23] System from 2014 to 2018 and were made available in anonymized DICOM format. The number of patients present in this dataset goes up to almost 14,000. Each case was annotated by expert radiologists, as was the DBT slice where the lesion is most visible. The patient IDs with the associated class, the available views, and the lesion slice information are also available at the referred website. The available data consist of three sets of images: training, validation, and testing. However, only the labels for the training set are available, so all of the data used in this study were derived from the training set. This set is composed of nearly 22,000 scans but not all of them were used. The rationale for the dataset constructed and used here was a balance amongst the different classes used, meaning that it was aimed to have approximately the same number of benign and malignant cases. Given that, the assembly of the dataset was limited by the class with the fewest observations. There were 39 available volumes of malignant images and, for that reason, 39 volumes from benign cases were also retrieved, making a total of 78 volumes considered for this work. As already mentioned, the goal was to make a single-slice classification and, for that reason, the single slice chosen was the one where the lesion was most visible. Thus, each volume contributed with a 2D slice that served to either train or test the developed model.

### 2.2. Image Preparation

Each of the 2D slices used in this study had to undergo a preprocessing methodology before being fed to the model. First, and following the information present in the dataset, all the images were segmented in order to retrieve small regions of interest (ROIs) that contained the lesions. For each DBT volume, experts annotated the slice where the lesion was most visible. Moreover, a small region that encapsulated the lesion was also defined. This information was used to manually extract the ROIs from each slice. After that, in order to improve image quality to ensure that the model was not misled, image adjustment techniques were applied to all ROIs. This technique aimed to improve the contrast of the image by saturating both the bottom and superior 1% of intensity values. Finally, the extracted regions of interest were normalized in terms of pixel intensity, so that the model did not learn by mistake random pixel intensity patterns that could be present in the images. A “min-max” normalization was applied (Equation (1)), which resulted in an image with pixel values ranging from 0 to 1. In the equation, *I* is the original image, min(I) is the overall minimum pixel intensity of image *I*, max(I) is the overall maximum pixel intensity of image *I*, and $I_{normalized}$ is the normalized image.

(1) $$I_{normalized}=\frac{I-min(I)}{max(I)-min(I)}$$

After that, each pixel value was multiplied by a scaling factor of 255. Given that each volume contributed with an image, 78 slices were available, which was not a substantial number of images for the proposed task. Actually, one of the malignant volumes was compromised so only 77 slices could be used. In order to overcome this problem, common data augmentation techniques were designed to increase both the variability and the number of available images. First, for each volume, instead of taking just the lesion slice, four slices above and four slices below the reference one were also extracted. The fact that the slices were retrieved from a relatively small neighbourhood of the lesion slice allowed us to guarantee that the lesion was still clearly visible, while still showing a slightly different breast configuration. On the other hand, these slices shared a lot of characteristics with the original lesion slice so the simple addition of them to the data pool could bias classification. With the purpose of surpassing this obstacle, an augmentation routine was applied three times to each of the slices. This routine consisted of randomly applying a transformation or a combination of transformations to each of the slices. The possible transformations defined were: a rotation ranging from −50° to 50°; a vertical translation ranging from 1 pixel to 5% of the vertical size of the image; a horizontal translation ranging from 1 pixel to 5% of the horizontal size of the image; a horizontal reflection; and a vertical reflection. The limits of the rotation and translation transformations were imposed with the rationale of guaranteeing that while the variation of the images was increased, the lesion remained within the image. Given the wide range of possibilities for the rotation transform and for both translations, there was great confidence that besides augmenting the number of available images, there was also considerable variability in the data used. This data augmentation procedure was done four times to each image, hence increasing four times the number of available 2D images. The original images and three of the four augmented images groups were used for training, while one of the augmented image groups was used for validation. Before feeding the images to the model, all of them were resized to dimensions of 224 × 224. Given that, 2772 (77 volumes × 9 slices × 3 augmentations + 77 volumes × 9 original slices) images were used for training and 693 images (77 volumes × 9 slices × 1 augmentation) were used for validation purposes. In order to test the model, the data augmentation routine was applied again, this time to the validation set, and predictions were made in this new augmented set of 693 images.

**Figure 1:**
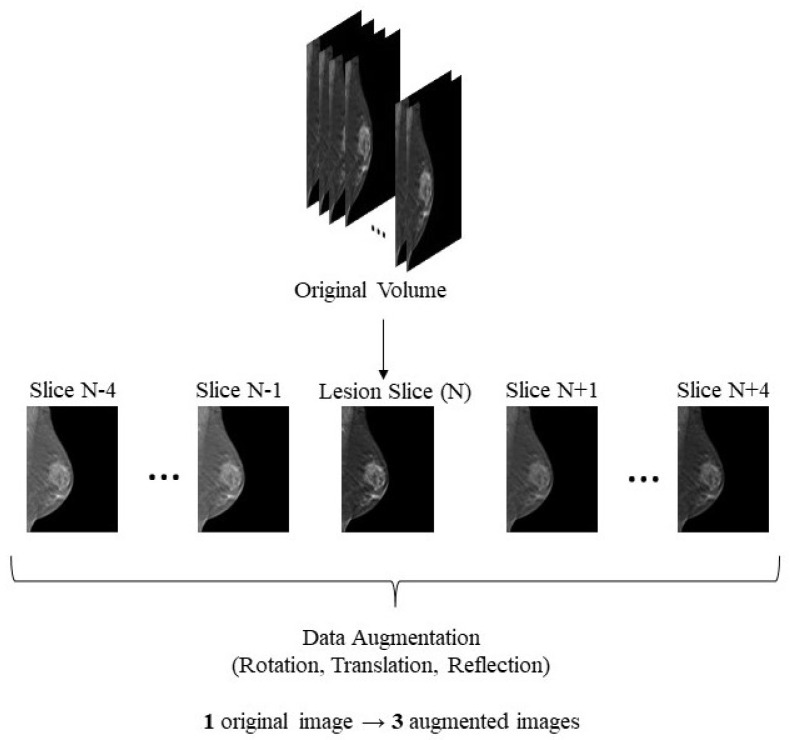
Data selection and augmentation—image contrast modified just for illustration purposes. From each original volume, the most representative slice and the four slices above and below were extracted. The rotation was randomly preformed in a range from −50° to 50°, the translation could be horizontal or vertical, ranging from 1 pixel to 5% of the overall image size, and the reflection could either be vertical or horizontal.

Figure 1 depicts the data selection and augmentation procedure for each DBT volume.

### 2.3. The Algorithm

As it was discussed during the introduction section, CNNs are widely used for the classification of medical imaging and, in particular, of DBT images. For that reason, a CNN model, inspired by the work of Muduli et al. [18], was developed from scratch. The created model was composed of five different layers: a convolution layer, an activation layer, a pooling layer, a fully connected layer, and a batch normalization layer. The convolutional layer is the base foundation of CNNs. These layers can be considered feature extractors that combine both linear operations (convolution) and nonlinear calculations (activation function). The convolution operation [24] is performed between a random array of numbers—kernel—and the input. Thus, given that the aim is to extract characteristics from the input, it can be said that the use of different kernels results in different feature extractors [25]. The result of this operation is, therefore, a feature map. When applying a convolution layer, one needs to predefine three convolution parameters: the kernel size, the number of filters, and the stride. The first one defines how much of the input the kernel sees at each convolution step; the second one has a self-explanatory name and defines the feature map depth; the stride, on the other hand, defines the space between two successive kernel positions during convolution. Moreover, it is known that the usual convolution operation results in an output feature map with a reduced size in comparison to the input. This is a problem if one wants to stack several sequential convolution layers—the size could become too small. Padding a neighbourhood of the image with pixels of intensity zero allows one to perform convolution operations while obtaining an output with the same size as the input.

In order to increase the relevance of the learned features, a nonlinear operation is applied to the feature map—an activation function. There are several activation functions but one of the most used is the rectified linear unit (ReLU) which takes in the input and outputs the maximum between the input and zero.

The pooling layer aims to reduce the dimensionality of the learnable parameters by summarizing the features present in a specific region of the feature map. This strategy, besides introducing the capability of learning invariant features, is also important for reducing the computational cost associated with a high number of parameters. For that reason, training time can also be reduced using pooling strategies [25].

A batch normalization layer, as the name implies, normalizes its input while aiming to maintain the activation values as stable as possible, so that it not only speeds up training but also provides good results. In addition, this layer also helps to avoid problems with overfitting [18].

Finally, fully connected layers usually receive as input the feature map derived from the last layer and aim to map this input to the last output of the network. It is possible to use several fully connected stacked layers and since in these layers, every input is connected to every output, the number of learning parameters can be highly increased. The output of the last fully connected layer is given as the input to the last activation function layer, where the chosen activation function needs to match the task that it is being aimed at; a softmax function is usually used.

**Figure 2:**
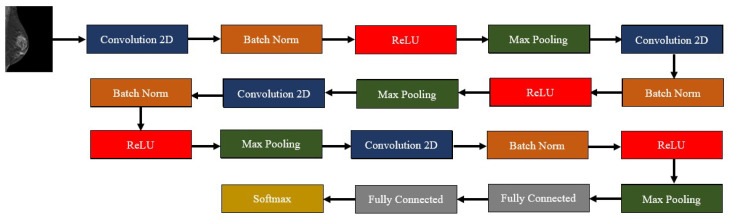
Outline of the developed deep learning algorithm—composed by four convolution–batch normalization–ReLU–max pooling blocks, followed by two fully connected layers and a softmax layer.

Our model was inspired by the one developed by Muduli et al., so it was composed of four convolution–batch normalization–ReLU–max pooling blocks. The max pooling parameters were always the same with a (2,2) pooling size and a stride of 2, and a padding routine that maintained the dimensions of the input in the output. On the other hand, the convolution layers increased the number of filters as the network deepened: 16, 32, 64, and 128 filters, respectively. The kernel size, however, decreased along the network: 9 × 9, 7 × 7, 5 × 5, and 3 × 3, respectively. After these blocks, two fully connected layers finalized the network, one with 128 units, and another one with 2—the number of classes of our dataset. The outputs of this last layer were mapped to a softmax layer. For learning, the Adam Optimizer [26] was used, with an initial learning rate of 0.001, the loss metric used was the sparse categorical cross-entropy, and the evaluation metric during training was the accuracy. As said, at each epoch, the model was evaluated using the previously created validation set; the training procedure continued until a maximum of 500 epochs unless the performance of the model achieved a baseline accuracy value (0.905) that did not further increase. In that case, the model stopped training before getting to the 500 epochs. In order to avoid overfitting, L2 (factor = 0.001) and dropout (factor = 0.2) regularizations were used. Between the fully connected layers the dropout factor was 0.5. Figure 2 generally depicts our model, while Table 1 describes each layer in the model.

## 3. Results

**Figure 3:**
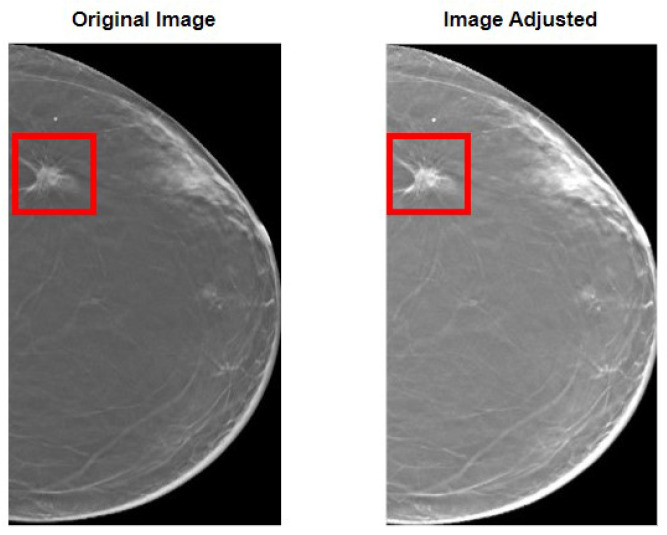
Results of the image adjustment procedure. On the left, the original image is shown with a bounding box encapsulating the lesion. On the right, the same can be seen but for the image after adjusting. It can be perceived how the lesion becomes more visible.

Our methodology started with the preparation of the images before feeding them to the model. First, in Figure 3, the overall results of the image adjustment in the entire image are shown, so that the effect of this methodology is fully noticed. As it can be perceived, the lesion becomes more visible, which is of extreme importance in the task of differentiating benign and malignant lesions.

**Figure 4:**
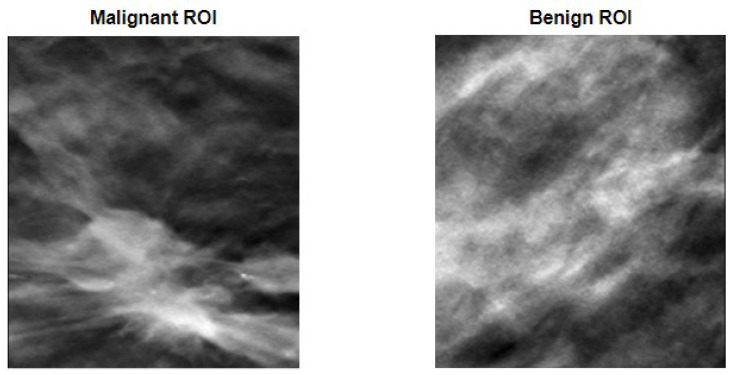
ROI definition to encapsulate malignant (**on the left**) and benign (**on the right**) lesions.

However, as said in the methodology section, rather than using the entire image of the breast, which could be a problem if the image had more than one lesion, ROIs were defined in a way that encompassed the lesion within their borders. Figure 4 shows two examples of defined ROIs, one for each of the considered classes.

**Figure 5:**
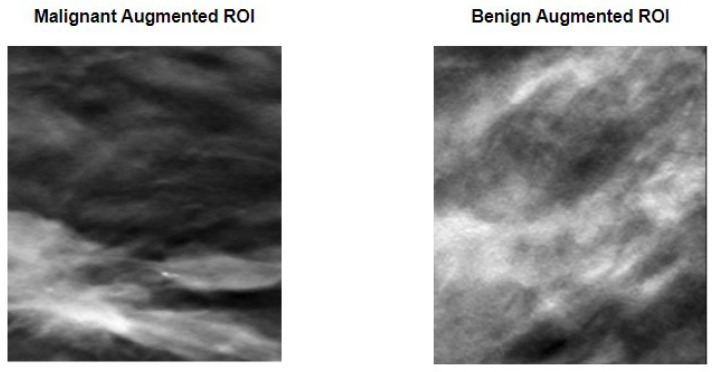
Data augmentation procedure applied to the previously seen ROIs. Random transformations that include rotation and/or translation and/or mirroring were applied to each ROI. As it can be perceived, the images are substantially different from what can be observed in Figure 4, while still maintaining the lesion within the image limits.

After the image preparation, and given the sparse number of available images to train the model, data augmentation techniques that consisted of random rotation, translations, and mirroring were performed. Figure 5 compares the two previously shown original ROIs with a version of the said images after a random transformation.

As it can be perceived, the images represent a clear distortion of the original images, while maintaining the lesions within the limits of the ROI. This fact means that the data augmentation technique used allowed us not only to increase the number and the variability of images available for training, but also to keep the lesions inside the defined ROI, hence not compromising the ground-truth labels given to the images. Table 2 specifies the number of images used for training, validation, and testing, and their division into the respective classes.

**Figure 6:**
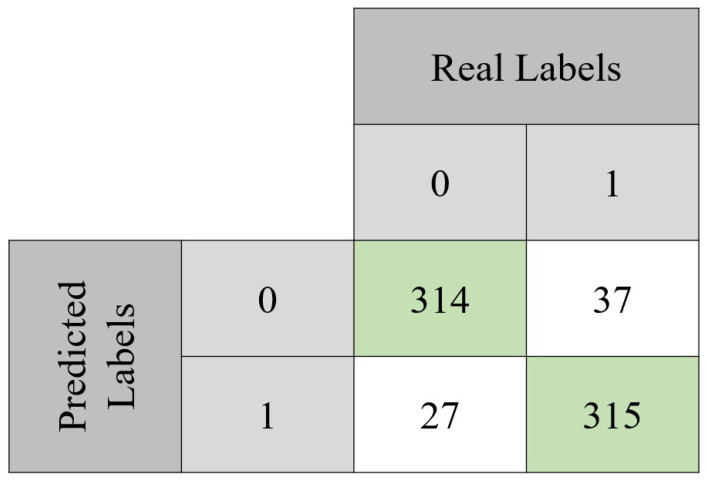
Confusion matrix obtained with the validation set. From the total 693 instances, the model was capable of correctly classify 629 of them. It can also be noted that approximately 92% of the benign lesions were correctly classified, while for the malignant lesions, the same happened for approximately 89% of the instances.

**Figure 7:**
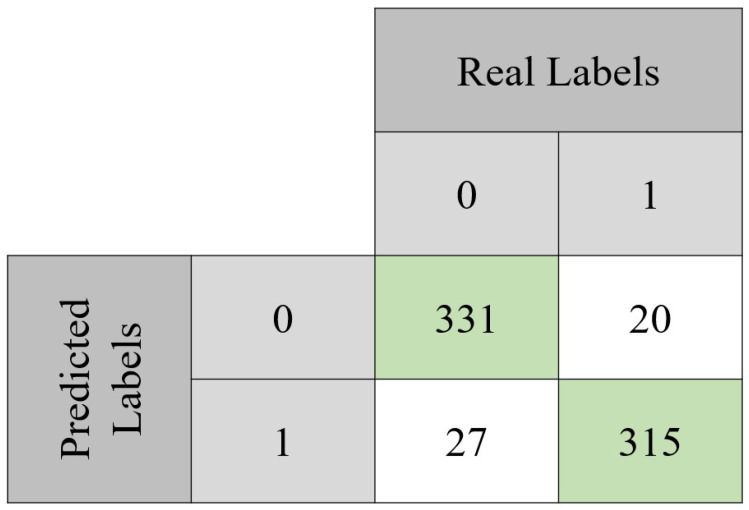
Confusion matrix obtained with the test set. From the total 693 instances, the model was capable of correctly classify 646 of them. It can also be noted that approximately 92% of the benign lesions were correctly classified, while for the malignant lesions, the same happened for approximately 94% of them.

The model was evaluated through classical performance metrics: accuracy, sensitivity, specificity, precision, and F1 score. Equations (2)–(6) show how to compute each one of these metrics. In order to compute them, the confusion matrix of both the validation set and the testing set is presented (Figure 6 and Figure 7). For the validation set, used to tune the model, the confusion matrix is seen in Figure 6. With those values, it was possible to compute the accuracy (90.7%) achieved after 494 epochs. Sensitivity, specificity, precision, and F1-score values were 92%, 89%, 89%, and 0.89 Regarding the testing set, the accuracy value was 93.2%. In terms of the previously mentioned metrics, their values in the testing set were 92%, 94%, 94%, and 0.94, respectively.

(2) $$Accuracy=\frac{Correct\;Cases}{All\;Cases}$$

(3) $$Sensitivity=\frac{True\;Positive}{True\;Positive+False\;Negative}$$

(4) $$Specificity=\frac{True\;Negative}{True\;Negative+False\;Positive}$$

(5) $$Precision=\frac{True\;Positive}{True\;Positive+False\;Positive}$$

(6) $$F1\;Score=\frac{2*Precision*Sensitivity}{Precision+Sensitivity}$$

## 4. Discussion

The goal of our study was to develop a CNN model that was able to differentiate malignant from benign lesions. In order to do that, ROIs that encapsulated lesions from the two classes were defined. These ROIs were then passed through an image enhancement algorithm that improved the contrast of the images with the goal of giving more visibility to the lesions, which was important in the context of differentiating the nature of the said lesions. As it was seen in the results section, the goal was achieved, as when comparing the original image of the breast with the image after the adjustment, the improvement of the image contrast was patent, specifically concerning the highlight of the lesions and their borders. After the ROI definition and image adjustment, data augmentation methodologies were employed with the aim of improving both the number of instances for model training and their variability. To do that, to each image in the dataset was applied a random transformation (or transformations) that consisted of rotations and/or translations and/or mirroring. Given the wide range of possible transformations and looking at the results shown in Figure 5, it is safe to say that the goal was accomplished. While the number of images was highly increased, having three variations for each original image on the dataset being used for training, the variability was also increased in a way that allowed the lesions to remain within the margins of the defined ROI. Having achieved the proposed goals in terms of image preparation, it was possible to look correctly at the results obtained in terms of training, validation, and testing of the proposed model. This model was trained from scratch, so, opposite to several of the papers reviewed in the introduction section, no transfer learning methodology was followed. The main metric used to assess model performance during training/validation was accuracy because it was the metric used by Muduli et al., a work close to what was aimed at here, with a very similar network. That being said, the results of the several metrics calculated both for the validation and the testing set were very positive and gave confidence on the robustness of the developed model. From the ten computed metrics, eight of them were above 90% and the remaining ones were not lower than 89%, which was extremely good. However, a good accuracy value by itself does not guarantee a good model. A model that arbitrarily defines all instances as zero can achieve a good accuracy in a scenario where only negative data are given to it. It was not the case in this study as the class balance was taken into account during dataset construction. The remaining metrics gave a higher sense of the robustness of the model; however, it would be interesting to have a metric that gives a clear indication of how much the predictions of the model agreed with the ground-truth labels. With that in mind, Cohen’s Kappa coefficient of agreement, that translates into a number of how much the agreement between two raters or readers is, was calculated. In the case of this paper, the “raters” were the ground-truth labels and the predictions made by the model. The calculation of this coefficient was done using Equation (7), where $p_{0}$ is the observational probability of agreement, and where $p_{e}$ is the expected probability of agreement [27]. Table 3 shows how to interpret this factor [27].

(7) $$kappa=\frac{p_{o}-p_{e}}{1-p_{e}}$$

The results for the validation and testing sets were of approximately 0.82 and 0.86, respectively, which according to Table 3 indicated an almost perfect agreement. All these results combined gave us confidence in the model that was able to correctly differentiate benign and malignant images from single slices of DBT images.

Most of the works reviewed in the introduction section used AUC as an evaluation metric, so a comparison between these studies and the work developed here is not fair. However, when looking at the obtained results, it is possible to say that our model achieved a good discriminatory capacity, which was proven by the F1 score and the kappa coefficient value. Thus, in that sense, it compared to most of the state-of-the-art papers reviewed in the introduction. For example, in the work presented in [12], it was found that the AlexNet models with transfer learning were the ones that presented better performance either for mammography (AUC = 0.7274) or DBT (AUC = 0.6632). As it can be perceived, a better performance was obtained with mammography than with DBT, which contradicted what was expected in theory; the authors pointed out that this may have been due to the fact the DBT volume was not entirely used and some of the discarded slices might have had relevant information. Our work, besides achieving a very good performance, tackled some of the problems that that research faced. On one hand, it used information present in the DBT volume from the most relevant slices and on the other hand, we used a 2D image that avoided the tissue overlapping present in mammography.

The authors of [16] compared the performance of their model with a classic AlexNet model but also compared, within their model, the use of a single imaging modality with the use of both modalities (FFDM + DBT). Their model, despite the use of a single or both modalities, outperformed 2D and 3D AlexNet. On the other hand, the use of an ensemble of imaging modalities outperformed the use of just DBT (AUCs of 0.97 and 0.89, respectively). A fair comparison between the work by these authors and the work done here is with their model using only DBT: for the four different backbone architectures, the accuracy values were of 81% (AlexNet), 79% (ResNet), 85% (DenseNet), and 79% (SqueezeNet). Our accuracy value on the testing set was 93.2%, which outperformed any of the models (the same happened for their models trained only on mammography). As for the F1-scores obtained by the authors, they ranged from 78 to 85%, which was lower than the value obtained in this work, 94%.

Zhang et al. [17] found that any of the variations of their proposed methodology (either late fusion of features, or a dynamic image) outperformed the classical architectures (3D convolution), which achieved 0.66 as the maximum AUC value. With their approach, they found out that the extraction of features from each slice and further pooling them into a final feature map produced better results than using the dynamic image, with maximum AUC values for each approach of 0.854 and 0.792, respectively, both using AlexNet architectures. Given the discrepancy in the metrics used, a fair comparison could not be made between their work and ours. However, their conclusions were interesting in that it showed the extraction of features from single slices not only outperformed the use of dynamic image but also classical 3D convolution approaches for DBT volumes.

On the other hand, a fairer comparison could be made between the developed work and the one presented by Muduli et al. These authors developed a model that could differentiate malignant and benign lesions on mammograms and ultrasound images. The architecture of the model was very similar to the one used here, differing in the number of fully connected layers and in the parameters (and type) of regularization. The comparison between the work done here and the one developed by Muduli’s team is important because it allows us to understand how this novel way of learning breast characteristics through a single slice of DBT can compare to the use of mammograms.

The three datasets of mammograms used by the authors achieved a performance, in terms of accuracy, of 96.55%, 90.68%, and 91.28%, on each of the three mammography datasets used. Considering our testing set, the model developed in this work reached an accuracy of 93%, which outperformed the model of Muduli in two of the three datasets used. In terms of sensitivity, our model was outperformed in two of the datasets used and was comparable to the other (92% vs. 97.28%, 92.72%, 99.43%). Finally, in terms of specificity, it is our model that outperformed Muduli’s work two out of three times (94% vs. 95.92%, 88.21%, 83.13%). As a result, overall, the model developed here was comparable and outperformed the use of mammograms, which indicates the potential that the use of single-slice DBT has in the field of AI applied to breast imaging.

Getting back to the works presented in the introduction section, the work proposed by Sun et al. [19] which consisted of an hybrid method with both 2D and 3D convolutions achieved an accuracy of nearly 80% while their F1 score was 83.54%. Both the results of this methodology, which used 2D convolutions for hierarchical feature representation and 3D convolutions for structural lesion information extraction, were outperformed by our methodology. A similar comparison could be made with the work of Xiao et al. [20], which had the same goal as the work of Sun et al. There, the best accuracy result achieved was 82%, while the F1 score obtained was 85.71%. As it happened with other research works, it was not fair to make a comparison between our work and the one done by [21] since different metrics were used; however, this study showed that the use of single DBT slices could yield very promising results.

However, it is important to analyse some flaws of the used methodology in order to improve future work or even to make it suitable for a real-life application. Originally, the dataset had 78 different 3D images before any strategy to augment the data. On the other hand, the datasets used by Muduli’s team had 326, 1500, and 410 different images. This discrepancy in original data, if overcome by the single-slice DBT annotated images, might result in outperforming the classical research based on the use of mammograms.

On the other hand, the use of this classic data augmentation should also be looked upon. While random rotations, translations, noise adding, or contrast variation help to increase variability and the number of instances used for training, it is important to understand, or to think about, how helpful they might be from the clinical point of view. Two transformed images from the same women are in fact different and surely contribute to the increase of variability in the dataset. However, these images are much closer to one another in terms of breast patterns than what happens in clinical practice for two images from different women. While the use of these augmentation techniques is important for the development of novel and better diagnostic models, different methods for increasing the quantity and the variability of data that are closer to real-world scenarios should be followed, such as the use of GANs [28].

Finally, there are several studies [12] that show how much transfer learning can help to improve the performance of the developed models while reducing the computational burden associated with training models. Here, we aimed to train the model completely from scratch, which not only resulted in an increased training time but might also negatively affect the performance of the model.

## 5. Conclusions

The work done throughout this study aimed to develop a CNN model that could classify breast medical images into two categories: malignant and benign lesions. While the used algorithm was inspired by another research work [18], we introduced the novel use of single-slice DBT for the classification. After extracting ROIs from the images on lesion locations and applying preprocessing methodologies to improve image quality, data augmentation techniques were performed to increase the size of the dataset while also increasing its variability. The results obtained in the several computed metrics showed that the performance of the developed model compared with state-of-the-art research and even overcame it in some cases. This fact suggests that the use of single slice DBT—which overcomes the problem with overlapping tissue present in mammograms—could play a major role once some of the identified flaws of this work are tackled. The information present in single slices is also present in the 3D volume that constitutes DBT. The rationale behind the use of a single-slice approach was based on a literary review where most of the architectures that used 2D convolutions outperformed the models that used a 3D approach. Therefore, it was with that in mind and considering the computational burden of developing from scratch a model using 3D convolution, that the single-slice approach was chosen in this work. While the reviewed works were either based on dynamic images or latent representations, we aimed to look for slices where the lesion was visible because, in one way, it had the planar characteristics of mammograms (allowing the use of 2D convolutions) and, on the other hand, the slice was so thin that it did not present the problem of overlapping tissue present in mammograms. This fact allowed us to diminish the possibilities of either lesion masking or the appearance of false positives, allowing the model to have a better grip on the proposed task. With that in mind, the following steps should be taken in future work: developing more real-life data augmentation techniques, inspired by what is seen in clinical practice in terms of breast configuration variability, and classifying whole mammograms/DBT instead of just one region of interest since it may help to find lesions that were overlooked. Furthermore, there is a great potential in transfer learning approaches, not only because it diminishes the computational burden of training a model (compared to a “from scratch approach“), but because it may also allow one to increase the performance of the developed models. The work developed in this paper served the purpose of showing how promising the use of single slices is for the differentiation between benign and malignant lesions. With that being established, further work should be focused on improving the performance of the models that are based on this approach. Transfer learning can play a major role in this task.

## Figures and Tables

**Table 1:** Layer-by-layer description for the developed model. As the model deepened, the number of filter increased (16-32-64-128) and the kernel size diminished (9-7-5-3). Max pooling had a stride and kernel size of 2 across the network. The numbers of units in the fully connected layers were 128 and 2.

Layer	#Filters (Conv) /Units (FC)	Kernel Size	Stride	Padding
Convolution—1	16	9	1	None
Batch Normalization	-	-	-	-
ReLU	-	-	-	-
Max Pooling	-	2	2	Same
Convolution—2	32	7	1	None
Batch Normalization	-	-	-	-
ReLU	-	-	-	-
Max Pooling	-	2	2	Same
Convolution—3	64	5	1	None
Batch Normalization	-	-	-	-
ReLU	-	-	-	-
Max Pooling	-	2	2	Same
Convolution—4	128	3	-	None
Batch Normalization	-	-	-	-
ReLU	-	-	-	-
Max Pooling	-	2	2	Same
Fully Connected	128	-	-	-
Fully Connected	2	-	-	-
Softmax	-	-	-	-

**Table 2:** Data division. Number of planar images used for training, validation, and testing.

	Training	Validation	Testing
**Benign**	1404	351	351
**Malignant**	1368	342	342

**Table 3:** Interpretation of kappa coefficient [27].

Coefficient Value	Strength of Agreement
<0.00	Poor
0.00–0.20	Slight
0.21–0.40	Fair
0.41–0.60	Moderate
0.61–0.80	Substantial
0.81–1.00	Almost perfect

## Data Availability

The dataset analyzed in this study can be found at https://wiki.cancerimagingarchive.net/pages/viewpage.action?pageId=64685580.
